# Inheritance of chloroplast and mitochondrial genomes in cucumber revealed by four reciprocal F_1_ hybrid combinations

**DOI:** 10.1038/s41598-021-81988-w

**Published:** 2021-01-28

**Authors:** Hyun-Seung Park, Won Kyung Lee, Sang-Choon Lee, Hyun Oh Lee, Ho Jun Joh, Jee Young Park, Sunggil Kim, Kihwan Song, Tae-Jin Yang

**Affiliations:** 1 Department of Agriculture, Forestry and Bioresources, Plant Genomics and Breeding Institute, College of Agriculture and Life Sciences, Seoul National University, 1 Gwanak-ro, Gwanak-gu, Seoul, 08826 Republic of Korea; 2 Phyzen Genomics Institute, Seongnam, Gyeonggi-do 13558 Republic of Korea; 3 Department of Horticulture, Chonnam National University, Gwangju, 61186 Republic of Korea; 4 Department of Bioresources Engineering, College of Life Sciences, Sejong University, Seoul, 05006 Republic of Korea

**Keywords:** Plant evolution, Plant sciences, Plant genetics, Comparative genomics, Genome evolution

## Abstract

Both genomes in chloroplasts and mitochondria of plant cell are usually inherited from maternal parent, with rare exceptions. To characterize the inheritance patterns of the organelle genomes in cucumber (*Cucumis sativus* var. *sativus*), two inbred lines and their reciprocal F_1_ hybrids were analyzed using an next generation whole genome sequencing data. Their complete chloroplast genome sequences were de novo assembled, and a single SNP was identified between the parental lines. Two reciprocal F_1_ hybrids have the same chloroplast genomes with their maternal parents. Meanwhile, 292 polymorphic sites were identified between mitochondrial genomes of the two parental lines, which showed the same genotypes with their paternal parents in the two reciprocal F_1_ hybrids, without any recombination. The inheritance patterns of the chloroplast and mitochondria genomes were also confirmed in four additional cucumber accessions and their six reciprocal F_1_ hybrids using molecular markers derived from the identified polymorphic sites. Taken together, our results indicate that the cucumber chloroplast genome is maternally inherited, as is typically observed in other plant species, whereas the large cucumber mitochondrial genome is paternally inherited. The combination of DNA markers derived from the chloroplast and mitochondrial genomes will provide a convenient system for purity test of F_1_ hybrid seeds in cucumber breeding.

## Introduction

Chloroplasts and mitochondria are essential plant organelles that perform photosynthesis and ATP generation, respectively, in addition to their involvement in the biosynthesis of compounds such as starch, oils, and amino acids^1^. While the nuclear genes show biparental Mendelian inheritance patterns, the chloroplast and mitochondrial genomes show non-Mendelian inheritance patterns, predominantly maternal inheritance^2^. This maternal inheritance pattern is probably a result of the high mutational load of the male gametes^2^. Alternative chloroplast and mitochondria inheritance patterns have been reported in some plant species however, making it difficult to understand the inheritance patterns of their organelles^2^.

Both the chloroplasts and the mitochondria in plants are believed to have evolved from ancient endosymbionts, and as such contain their own genomic material. While the chloroplast genome structure and size are maintained around 120–160 kb^3–13^, plant mitochondrial genome structure and size are highly dynamic, including several forms and sizes in the range of 220–3000 kb^14–16^. This variation contrasts with the animal mitochondrial genome, which is a highly compact, simple, and intact structure, comprising approximately 16 kb of circular DNA^17^.

Cucumber (*Cucumis sativus* var. *sativus*) is one of the most widely cultivated and economically important fruit crops in the world^18,19^. In addition, cucumber is a model plant for the study of sex determination and vascular biology^20,21^; therefore, many cucumber cultivars and wild cucumber species have been subjected to analyses of their nuclear and organellar genomes^22–30^. These studies have shown that cucumber has a narrow genetic diversity, which makes the breeding of this species more difficult^19^. To date, eight cucumber chloroplast genome sequences have been reported^31^. By contrast, the complete mitochondrial genome sequence has only been reported for the cucumber cultivar Calypso^32^ and was found to comprise three chromosomes, including a major larger chromosome (~ 1.6 Mb) and two smaller chromosomes (84 kb and 45 kb each). The cucumber mitochondrial genome is almost 10 times longer than that of its chloroplast genome (~ 160 kb).

Analyses of the polymorphism patterns present in small regions of the cucumber organelle genomes have revealed that the chloroplast and mitochondrial genomes of this species are inherited maternally and paternally, respectively^33–36^. Understanding these inheritance patterns is particularly important because some key cucumber traits are closely associated with the organelles and their inheritance; for example, the cucumber chilling temperature response was reported to be maternally or paternally inherited, depending on the genetic background, implying that it could be under the control of a factor encoded by an organelle genome^23^. In addition, cytoplasmic male sterility and variegated phenotypes have been linked to the mitochondrial genome in plant species such as radish (*Raphanus sativus*), pepper (*Capsicum annuum*), *Arabidopsis thaliana*, and tobacco (*Nicotiana tabacum*)^16,37–39^.

Next-generation sequencing (NGS) techniques enable the simultaneous assembly of the chloroplast and mitochondrial genome sequences at a low cost, which has facilitated the sequencing of a large number of plant organelle genomes, enabling their in-depth study^3,4,8^. In this study, we tried to clarify the inheritance pattern of chloroplast and mitochondrial genomes based on NGS whole-genome sequencing data using several cucumber parental lines and their reciprocal F_1_ hybrids.

## Results

### Complete chloroplast genomes of two parental inbred lines and their F_1_ hybrids

**Figure 1:**
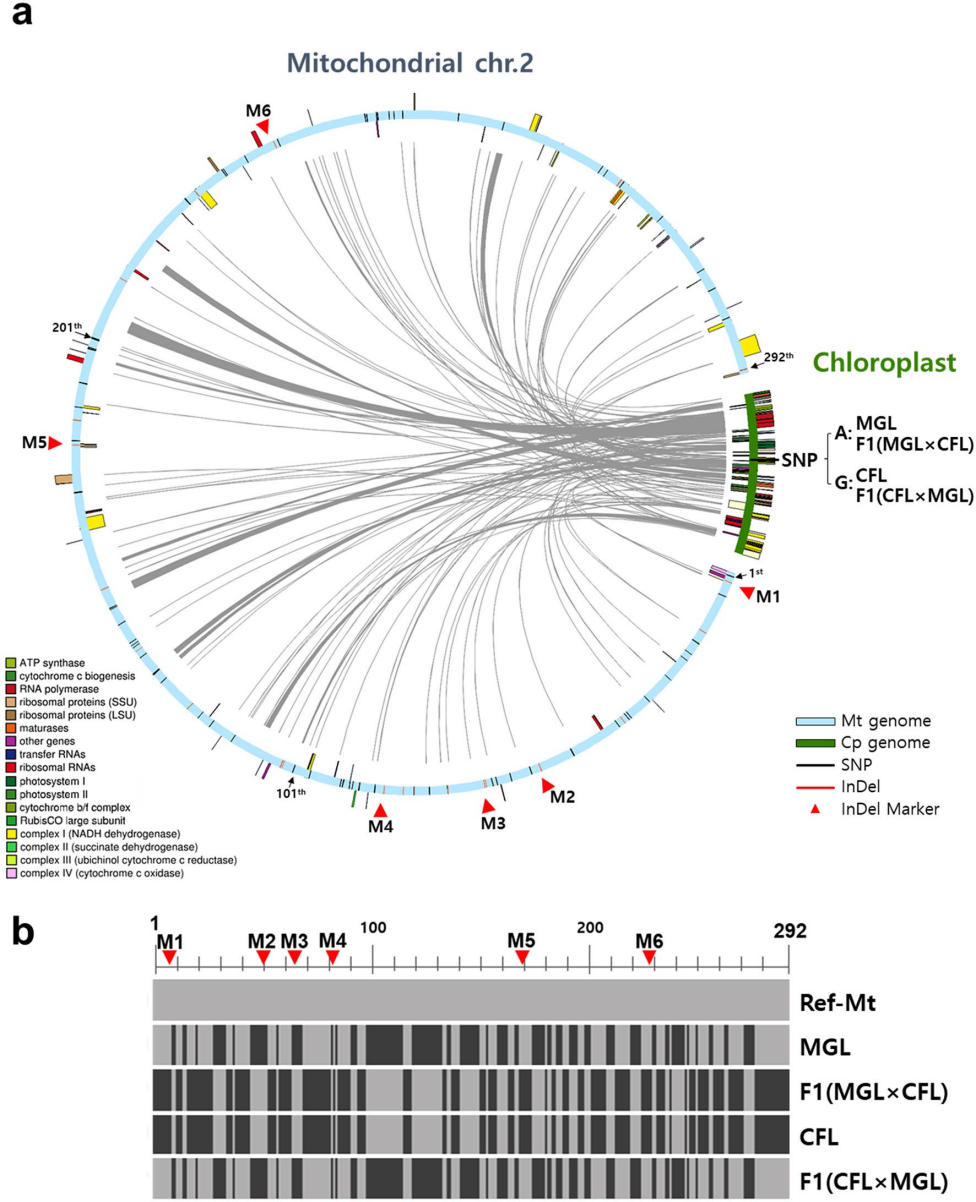
Schematic representation of mitochondrial and chloroplast genome of *Cucumis sativus*. (**a**) Each colored bar indicates the mitochondrial chromosome 2 retrieved from NCBI (NC_016005.1) and chloroplast genome of the inbred line MGL in this study. One of the pair of the inverted repeat regions in chloroplast genome was removed. Genes transcribed clockwise and counterclockwise are located on the outside and inside of the bar, respectively. The 292 mitochondrial and 1 chloroplast polymorphisms between two parental lines, MGL and CFL, are represented as black lines for SNP and red lines for InDels. The mitochondrial plastid DNAs and their plastid origins are linked with gray line. The positions of six InDel markers (M1 to M6) are labeled with red triangles. (**b**) Genotype comparison of the 292 polymorphic sites in mitochondrial genome of parental lines and F_1_ hybrids of reciprocal cross. The same color indicates the same genotype for polymorphic sites among parental lines and F_1_ hybrids. Gray-mitochondrial indicates the genotype of the reference mitochondrial genome (NC_016005.1).

A total of 3.8 Gb PE reads (about 0.9–1.0 Gb for each of the four cucumber samples) were obtained and quality-trimmed (Table 1). The resulting high-quality PE reads (670–800 Mb) from each sample were independently de novo assembled to generate the complete chloroplast genome sequences of two cucumber parental lines, MGL and CFL, and their two reciprocal F_1_ hybrids, MGL × CFL and CFL × MGL. All the chloroplast genomes had the same size, 155,525 bp, and possessed a typical quadripartite structure, consisting of a large single-copy (LSC) region of 86,877 bp, a small single-copy (SSC) region of 18,274 bp, and a pair of inverted repeats (IRa and IRb) comprising 25,187 bp (Fig. 1, Supplementary Figs. S1 and S2, and Table S1). All of the chloroplast genomes contained a total of 120 genes, including 79 protein-coding genes, 37 tRNA genes, and four rRNA genes. The average depths of the trimmed NGS data mapped to the complete chloroplast genome sequences ranged from 540× to 690×. The complete chloroplast genome sequences of the two parental inbred lines and their two reciprocal F_1_ hybrids were deposited in GenBank under the accession numbers KX231327, KX231328, KX231329, and KX231330, respectively.

**Table 1:** Cucumber samples and NGS data used in this study.

Cucumber samples	Generation	Raw data		Trimmed data		NABIC accession nos
		Reads	Bases	Reads	Bases	
MGL^a^	Parent	2,861,968	856,903,647	2,404,700 (84.0%)	568,007,755 (66.3%)	NN-4028
CFL^b^	Parent	3,110,572	931,526,838	2,655,587 (85.4%)	628,169,399 (67.4%)	NN-4031
F1 (MGL × CFL)	F_1_ hybrid	3,388,454	1,014,964,305	2,873,832 (84.8%)	679,680,316 (67.0%)	NN-4030
F1 (CFL × MGL)	F_1_ hybrid	3,191,966	955,434,149	2,758,084 (86.4%)	654,654,520 (68.5%)	NN-4032

^a^Korean solid green type inbred line.

^b^Chinese long green type inbred line.

The only one SNP was identified between the chloroplast genomes of the two parental inbred lines MGL and CFL, which was located at position 53,610 bp in the intergenic region between *ndhC* and *trnV-UAC* (Fig. 1a). A comparison of our chloroplast genome sequences with those reported for other cucumber cultivars [GY14 (DQ865975), CHIPPER (DQ865976), Baekmibaekdadagi (DQ119058), Borszczagowski (AJ970307), and hardwickii isolate PI183967 (MF536709)] (Supplementary Table S2) revealed very low-level diversity (1 to 39 SNPs and 3 InDels) among korean breeding lines and a few dozen SNPs and InDels (50 to 288 SNPs and 19 to 150 InDels) between the wild collections or the North-European cucumber (Borszczagowski, AJ970307).

### Polymorphisms in the mitochondrial sequences of the two parental inbred lines

Since the cucumber mitochondrial genome is huge (~ 1.6 Mb in NC_016005), the complete mitochondrial genome could not be assembled using the short NGS reads generated in this study. Instead, the polymorphic sites, SNPs, and InDels were investigated using NGS read mapping. A total of 292 polymorphic sites, including 246 SNPs and 46 InDels, were identified between the mitochondrial sequences of the two parental inbred lines (Fig. 1b, Supplementary Table S3). The NGS mitochondrial reads mapped onto the polymorphic sites had an average coverage of 22.7×. Among the 272 polymorphic sites, 240, 46, and six were identified in intergenic regions, introns, and exons, respectively. The six exonic SNPs were identified in three genes; *rps1*, encoding a ribosomal protein; *ccmB*, encoding an ABC transporter subunit; and *nad7*, encoding a NADH dehydrogenase subunit. All six exonic SNPs were non-synonymous substitutions that resulted in amino acid changes.

### Validation of chloroplast and mitochondrial genome inheritance using DNA markers

Molecular markers were developed based on the polymorphic sites identified in the chloroplast and mitochondrial sequences. For the chloroplast sequence, a pair of dCAPS markers were designed based on the single SNP identified between the chloroplast genomes of the two parental inbred lines (Fig. 1a). For the mitochondrial sequence, six targets with more than a 20-bp InDel between the two parental lines were selected among the 292 polymorphic sites (Fig. 1b, Table 2). Overall, seven polymorphic sites, one from the chloroplast genome and six from the mitochondrial genome, were used to design molecular markers for the investigation of the inheritance patterns of the chloroplasts and mitochondria (Table 3).

**Table 2:** InDel sites and sequences with length difference of more than 20 bp in mt sequences between two parental lines.

Position^a^	Intergenic region location^b^	Consensus	Reference^c^	MGL	CFL	F1 (MGL × CFL)	F1 (CFL × MGL)	Developed marker ID
7,613	*ccmFc-nad5*	GTCCTACTTCCATATGGATA	–	–	InDel1 (20 bp)	InDel1 (20 bp)	–	Mt-InDel-01
226,850	*rrnS-rps7*	CTACGGATAAATCCTCCGGA	TR20 (20 bp)	–	TR20 (20 bp)	TR20 (20 bp)	–	Mt-InDel-02
274,021	*rps7-trnH-GUG*	GTAGGACTATCCATTAGAA	InDel3 (20 bp)	–	InDel3 (20 bp)	InDel3 (20 bp)	–	Mt-InDel-03
355,356	*rps7-trnH-GUG*	CTACTTCTAATGGATAGTC	InDel4 (19 bp)	–	InDel4 (19 bp)	InDel4 (19 bp)	–	Mt-InDel-04
766,753	*rps4-nad6*	TTATCCGTAGTCCGGAGGAT	–	–	InDel5 (21 bp)	InDel5 (21 bp)	–	Mt-InDel-05
1,074,192	*rrnL-trnP*	CTACGGATAAATCCTCCGGA	–	–	TR20 (20 bp)	TR20 (20 bp)	–	Mt-InDel-06

^a,b,c^Based on mt genome sequence (NC_016005.1, 1,555,935 bp) previously reported in cucumber cultivar Calypso^32^.

“–”, is representing non-detected indels or tandem repeats.

**Table 3:** Molecular markers designed in this study.

Marker ID	Marker type	Direction	Primer sequences (5′-3′)	Estimated amplicon sizes (bp)				Genotyping^e^
				MGL	CFL	F1(MGLx CFL)	F1(CFL × MGL)	
Cp-SNP-01	dCAPS (*Eco*RV)	F	GGTGGAGTTTTTAACCGGTTT**G**AT^a^	269 (245^b^)	269 (269^b^)	269 (245^b^)	269 (269^b^)	Cp
		R	TTCGAGTCCGTATAGCCCTATATTA					
Cp-SNP-02	dCAPS (*Rsa*I)	F	TGAAGAAGGAAAGAGAAGACGACTA	256 (256^d^)	256 (231^d^)	256 (256^d^)	256 (231^d^)	Cp
		R	GTAAATTTTGGGAGTCAAAATCA**GT**A^c^					
Mt-InDel-01	InDel	F	GCGGACTGCTTCGGTTAAT	258	265	265	258	Mt
		R	ACCTGTCCTACGTGCCAAAG					
Mt-InDel-02	InDel	F	AGAAAGGTCCAAAGCCCTTC	329	354	354	329	Mt
		R	GCAGCGAGAAAGTCAGAGGA					
Mt-InDel-03	InDel	F	TTCTCTCCGTACCGCGTAGT	347	371	371	347	Mt
		R	TCACCTGGACCTTTCTTTGG					
Mt-InDel-04	InDel	F	ACCTTAGGCCGGACCTCTTA	365	389	389	365	Mt
		R	CATCCATATGGGCCAAAGAA					
Mt-InDel-05	InDel	F	TTAGAAGCAGTCCGCAGGAT	303	328	328	303	Mt
		R	ACCGTCCGTAGGAGTCCTTT					
Mt-InDel-06	InDel	F	CCGGGAAATAGCAATGAAAG	555	580	580	555	Mt
		R	ACCTTCCTGCTTCAGGGAAT					
Nu-InDel-01	InDel	F	GCTTGAATTTGGGCAACAGGT	332	373	332/373	332/373	Nucleus
		R	TCTTGAAGTGGAGGTGTCCG					
Nu-InDel-02	InDel	F	CCACTGATGACAATGCCTGT	309	264	309/264	309/264	Nucleus
		R	ACGAAATTTTCTGTAACTCATGTAA					

^a,c^Bold letters indicate modified bases for cleavage of PCR amplicon by restriction enzymes (*Eco*RV and *Rsa*I).

^b,d^Fragment size after cleavage of PCR amplicon by restriction enzymes for each dCAPS marker.

^e^Cp indicating chloroplast and Mt indicating mitochondria.

**Figure 2:**
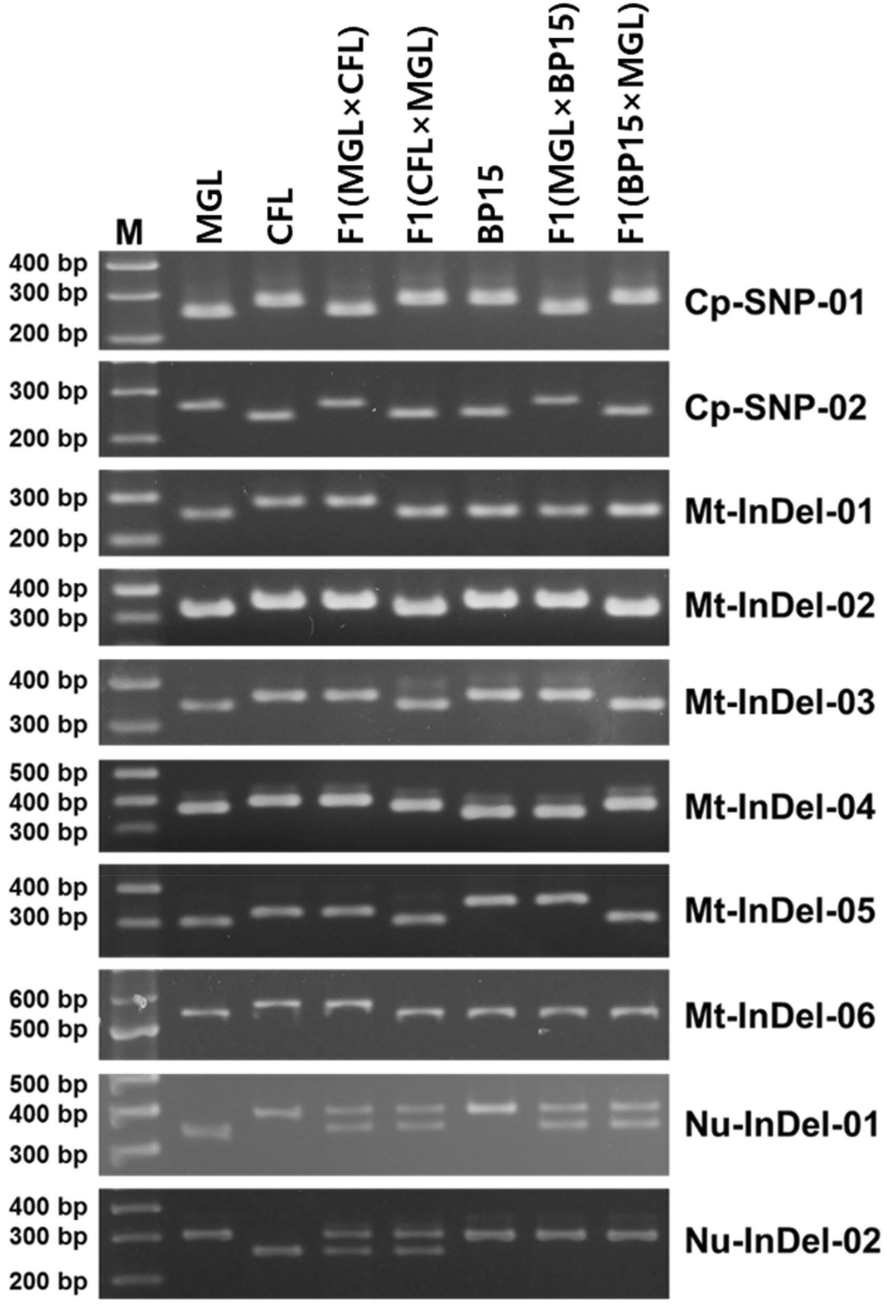
Validation of molecular markers to confirm inheritance pattern of organelles in cucumber. Eight molecular markers (Cp-SNP-01 to Mt-InDel-06) were designed based on chloroplast and mitochondrial sequence polymorphisms and validated using genomic DNA PCR analyses with seven cucumber samples. Two nuclear InDel markers, Nu-InDel-01 and Nu-InDel-02, were used to confirm heterozygous genotypes of F_1_ hybrid nuclear genomes. M, 100-bp size marker; MGL, a Korean solid green-type inbred line; CFL, a Chinese long green-type inbred line; F1(MGLxCFL), an F_1_ hybrid between MGL (maternal) and CFL (paternal); F1(CFLxMGL), an F_1_ hybrid between CFL (maternal) and MGL (paternal); BP15, a Beith alpha-type inbred line; F1(MGLxBP15), an F_1_ hybrid between MGL (maternal) and BP15 (paternal); F1(BP15xMGL), an F_1_ hybrid between BP15 (maternal) and MGL (paternal).

The molecular markers were used for genotyping of the two parental inbred lines and their two reciprocal F_1_ hybrids. The chloroplast-derived markers revealed that the F_1_ hybrids shared an identical genotype with their maternal parent in each cross (Fig. 2). By contrast, all six of the mitochondria-derived markers were used to reveal identical genotypes between the F_1_ hybrids and their paternal parent (Fig. 2).

**Figure 3:**
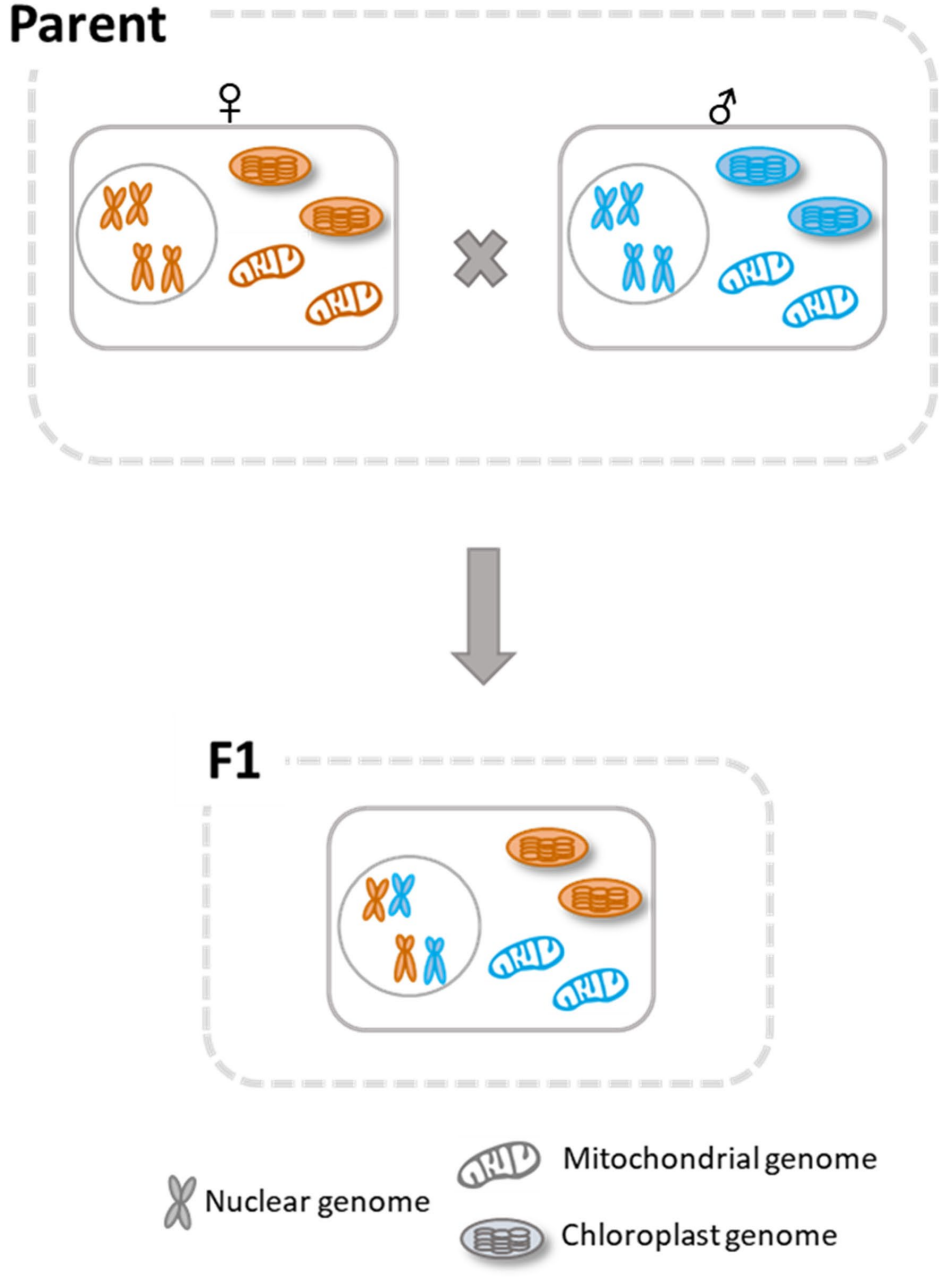
Summarized inheritance patterns of chloroplast and mitochondria in cucumber. Organelle inheritance from parental inbred lines to their two reciprocal F_1_ progeny was elucidated through whole-organelle genome- and polymorphic marker-based genotyping, confirming that cucumber chloroplasts were inherited maternally while mitochondria were inherited paternally. Colors indicate genotypes.

The inheritance patterns of the chloroplast and mitochondrial markers were also validated in other cucumber parental inbred lines (BP15, YHB, HHG, and KWS) and their F_1_ hybrid plants (Fig. 2, Supplementary Fig. S3). The maternal inheritance of chloroplast genome and paternal inheritance of mitochondrial genome were confirmed from all six reciprocal crosses of the four parental lines. The heterozygous genotypes of the nuclear genomes in the F_1_ hybrids were confirmed by genotyping the two nuclear InDel markers, Nu-InDel-01 and Nu-InDel-02 (Table 3, Fig. 3, Supplementary Fig. S3), which were designed based on the InDel polymorphisms identified in the intron of the cucumber gene *Csa1G042170* on chromosome 1 and the 3′ UTR of the cucumber gene *Csa3G127780* on chromosome 3.

## Discussion

### Reconfiguration of the organellar genome inheritance pattern in cucumber using NGS sequencing

Here, we generated approximately 1 Gb of NGS data, which is about three-fold the haploid genome coverage of *Cucumis sativus*^24^. These data were used to assess the genetic diversity and inheritance of the organellar genomes. This quantity of data was sufficient to facilitate the assembly of the chloroplast genomes of the four assessed cucumber lines, which confirmed their maternal inheritance based on the inheritance of one SNP identified between the parental lines. We also identified 246 SNPs and 46 InDels across the mitochondrial genome by mapping NGS reads directly to the reference cucumber mitochondrial genome.

Here, we used complete genome sequences to validate the previously reported inheritance patterns of the organellar genomes, which were identified using only a few Restriction Fragment Length Polymorphism (RFLP) markers^33,34^. This inheritance pattern was further inspected in the present study using another four cucumber lines and their reciprocal F_1_ progenies. Our method is based on advanced NGS technologies and could be widely used not only for the study of the inheritance patterns of plant organellar genomes, but also for the study of mitochondrial genomes in general, which are usually more difficult to fully assemble than the chloroplast genomes.

### Chloroplast genome diversity is low in cucumber, while its mitochondrial genomes are more diverse

In this study, the chloroplast genomes of the four cucumber lines (MGL, CFL, their reciprocal F_1_ hybrids) could be fully assembled; however, the mitochondria genomes could not. This is not only because the chloroplast copy number is generally much higher than that of the mitochondria, but also because the cucumber mitochondrial genome is unusually large and complex^32^.

The mitochondrial gene sequences are more conserved than the chloroplast genes^40,41^; however, here, we identified 52 polymorphic sites in the mitochondrial gene regions between two cucumber lines but none in the chloroplast gene regions. The six exonic polymorphic sites in the mitochondrial genome were non-synonymous mutations, which caused amino acid changes in the corresponding protein sequence. Genes containing the non-synonymous mutations included *rps1*, encoding a ribosomal protein; *ccmB*, encoding an ABC transporter subunit; and *nad7*, encoding a NADH dehydrogenase subunit. In Arabidopsis, the chloroplast *rps1* gene is involved in heat stress tolerance^42^, and may help to optimize chloroplast integrity under heat stress. It is therefore possible that the cucumber *rps1* gene in the mitochondrial genome might also be involved in the heat stress response. The mitochondrial *ccmB* is involved in cytochrome c and c1 biogenesis in wheat^43^. *Nad7* encodes a NADH dehydrogenase, which is involved in the essential respiratory chain. The inhibition of the respiration is a main cause of the production of reactive oxygen species, which can damage cells and tissues^44^. The important roles these genes play in plants suggests that future studies should explore how their identified mutations in cucumber affect the phenotypes of these plants.

### The rich mitochondrial genome diversity may be caused by its paternal inheritance pattern in cucumber

Many plastid-derived DNA fragments have been identified in plant mitochondrial genomes^45,46^. The total length of this mitochondrial plastid DNA (MTPT) in cucumber is 69 kb, which is the highest amount of MTPT among the mitochondrial genome sequences reported to date^46^ (Supplementary Fig. 3). At 1.6 Mbp, the cucumber mitochondrial genome is one of the biggest in plants and is almost six times larger than the smallest plant mitochondrial genome, which is around 220 kbp in *Brassica* species^14^. As discussed above, the cucumber mitochondrial genes are particularly diverse relative to the diversity observed in other plants^41^, and MTPT is also abundant in the cucumber mitochondrial genome. We can therefore assume that the abundant gene diversity in the cucumber mitochondrial genome is related to its paternal inheritance trait and its high content of MTPT fragments. Similar cases were reported in the organelle genomes of gymnosperms including the Pinaceae and Taxaceae, which are often paternally inherited^47^. The synonymous substitution rates of both chloroplast and mitochondrial genomes were reported to be higher in species displaying paternal inheritance rather than maternal inheritance^48^.

In animals, the organellar genomes are rarely inherited paternally or biparentally^49^; however, plants with unique organelle inheritance patterns are not as rare as their animal counterparts. Most conifers display paternal chloroplast inheritance patterns^50,51^, while the chloroplast genomes of alfalfa (*Medicago sativa*) and *Oenothera* spp. are biparentally inherited^2^. Like cucumber, wild bananas (*Musa acuminata*) display a maternal chloroplast inheritance and a paternal mitochondrial inheritance^52^.

### DNA markers derived from the chloroplast and mitochondria genomes can be used to assess the genotypes of F_1_ hybrid seeds

Ten markers designed to target polymorphisms in the mitochondrial, chloroplast, and nuclear genomes were successfully applied to validate the genomic inheritance patterns in the F_1_ progenies derived from crosses between several cucumber lines.

Plant cytoplasmic organelles are not only involved in photosynthesis and respiration; recent studies have revealed their diverse roles, including in agriculturally important traits such as male sterility^16,53,54^. The cucumber mitochondrial genes may also affect male sterility properties, making the paternal inheritance of the cucumber mitochondrial genome especially important for agriculture. The independent inheritance of the cucumber chloroplasts and mitochondria must be considered in breeding programs. The genome sequences and markers developed here are therefore expected to be of great value in the cucumber breeding industry. In addition, we expect that our results could be used as a foundation to further the research into plants with unusual organelle inheritance traits, particularly in cucumber itself. The molecular markers developed in this research could be practically applied to the genotyping of other cucumber inbred lines or samples; for example, combinations of the chloroplast and mitochondrial markers could be used to check the purity of F_1_ hybrid cucumber seeds as they are easily detected high-copy targets derived from the maternal and paternal parents, respectively.

## Materials and methods

### Plant materials

Two cucumber parental inbred lines, MGL bred from Korean solid green-type cucumber and CFL bred from Chinese long green-type cucumber, and their two reciprocal F_1_ hybrids, MGL × CFL and CFL × MGL, were subjected to whole-genome sequencing using NGS technology. The additional inbred lines BP15, HHG, YHB, and KWS, as well as their F_1_ hybrids, were also used for the molecular marker tests. All the breeding lines and reciprocal F_1_ hybrids were developed in this study.

### Whole-genome sequencing of cucumber

Total genomic DNAs were extracted from fresh leaves using a modified cetyltrimethyl ammonium bromide (CTAB) method^55^ and their quality was examined using agarose electrophoresis and a spectrometer. Paired-end (PE) libraries with a 300-bp insert size were constructed according to the standard Illumina PE protocol, and the pooled PE libraries were sequenced by LabGenomics (http://www.labgenomics.co.kr, Seongnam, Republic of Korea) using the HiSeq2000 platform (Illumina, USA). The sequencing data were deposited in the National Agricultural Biotechnology Information Center (NABIC, http://nabic.rda.go.kr)^56^.

### Assembly and comparison of the complete chloroplast genome sequences

The PE reads were quality-trimmed using the CLC quality trim tool with default parameters, which is included in the CLC ASSEMBLY CELL package (ver. 4.6 beta; CLC Inc., Denmark; http://www.clcbio.com/products/clc-assembly-cell/). Afterward, high-quality PE reads (Phred scores > 20) were de novo assembled using the CLC genome assembler included in the package, as described previously^3,4^. The chloroplast genome contigs were extracted, ordered, and merged to generate a single draft sequence, based on the reported reference chloroplast genome sequence of *C. sativus* (DQ865975; Gy14 cultivar; 155,525 bp)^23^. The draft chloroplast sequences were manually combined, corrected, and gap-filled using a series of PE read mapping. A single ambiguous sequence caused by low coverage of PE read mapping was found only in F1 (MGL × CFL) and confirmed using genomic PCR amplification and nucleotide sequencing. The chloroplast genome was annotated using GESEQ (https://chlorobox.mpimp-golm.mpg.de/geseq.html), BLAST searches, and a comparison with the reference cucumber chloroplast genomes. The chloroplast genome sequence of each sample was independently assembled.

Sequence polymorphisms were identified by a comparison of the chloroplast genomes in the two parental inbred lines and their F_1_ hybrids using multiple sequence alignment tools such as ClustalW (http://www.genome.jp/tools/clustalw/), MAFFT ver. 7 (http://mafft.cbrc.jp/alignment/server/index.html), and a BLAST-based alignment (https://blast.ncbi.nlm.nih.gov/Blast.cgi).

### Investigation of polymorphic sites in the mitochondrial genome sequences

High-quality PE reads of each of the four cucumber samples were mapped to the previously reported reference mitochondrial genome sequence (NC_016005; 1,555,935 bp) from the cucumber cultivar Calypso^32^ using BWA ver. 0.7.10 (http://bio-bwa.sourceforge.net) with default parameters. Positions of properly mapped reads were selected using SAMtools ver. 1.1 (http://samtools.sourceforge.net/) and in-house scripts. Polymorphic sites [SNPs and insertion and deletion (InDel) mutations] were identified using Picard ver. 1.112 (http://broadinstitute.github.io/picard/) and GATK ver. 3.1 (https://www.broadinstitute.org/gatk/) with default parameters. All procedures were performed by Phyzen (http://www.phyzen.com/; Seongnam, Republic of Korea).

### Development and validation of molecular markers

Polymorphic sites identified in the chloroplast and mitochondrial sequences were used to design molecular markers for the analysis of the chloroplast and mitochondrial genotypes in other cucumber lines. PCR primers were designed to target the polymorphic regions using the NCBI Primer-BLAST tool (https://www.ncbi.nlm.nih.gov/tools/primer-blast/) for InDel markers and the dCAPS Finder 2.0 (http://helix.wustl.edu/dcaps/dcaps.html) for derived cleaved amplified polymorphic sequence (dCAPS) markers.

A PCR amplification with 2–50 ng genomic DNA templates was used to validate the markers. The PCR consisted of 25–30 cycles of 95 °C for 1 min, 52–65 °C for 1 min, and 72 °C for 1 min. For the optimal amplification of each specific PCR product, the cycles and annealing temperatures of the PCR reactions were adjusted for each of the markers. A final concentration of 1.0–1.5 M betaine was also used to enhance the specificity of the amplification. For the dCAPS marker, the amplified PCR products were digested with the corresponding restriction enzymes for 24 h. The PCR products or cleaved PCR products were separated on a 1.5–3.0% agarose gel containing ethidium bromide and visualized using an UV illuminator. All of the uncropped gel image was included in Supplementary information (Supplementary Fig. S4 and S5).

In addition, InDel polymorphic sites in the nuclear genome sequences of the two parental lines were identified by mapping the NGS reads to the cucumber reference nuclear genome sequence (v2.0; http://www.icugi.org/cgi-bin/ICuGI/index.cgi)^24^ using the same method described for the mitochondrial genome sequence. These nuclear InDel sites were then used to design markers to validate the heterozygous nuclear genotype of the F_1_ hybrids.

## Supplementary Information

Supplementary Figures.

Supplementary Tables.

## Data Availability

The sequencing data were deposited in the National Agricultural Biotechnology Information Center (NABIC, http://nabic.rda.go.kr) with accession numbers of NN-4028, NN-4031, NN-4030, NN-4032. The four of chloroplast genomes of *C. sativus* were deposited in NCBI Nucleotide Database with accession numbers of KX231327, KX231328, KX231329, and KX231330.
