# Supplementary material for: Inheritance of chloroplast and mitochondrial genomes in cucumber revealed by four reciprocal F1 hybrid combinations
Source: Sci Rep. 2021 Jan 28;11:2506. doi: 10.1038/s41598-021-81988-w (PMC7843999; doi:10.1038/s41598-021-81988-w)
Supplement: Supplementary file 1 — Supplementary Figures. [file 41598_2021_81988_MOESM1_ESM.docx]

**Inheritance of chloroplast and mitochondrial genomes in cucumber revealed by four reciprocal F_1_ hybrid combinations**

Hyun-Seung Park^1+^, Won Kyung Lee^1+^, Sang-Choon Lee^2^, Hyun Oh Lee^2^, Ho Jun Joh^1^, Jee Young Park^1^, Sunggil Kim^3^, Kihwan Song^4*^, and Tae-Jin Yang^1*^

^1^Department of Agriculture, Forestry and Bioresources, Plant Genomics & Breeding Institute, College of Agriculture & Life Sciences, Seoul National University, 1 Gwanak-ro, Gwanak-gu, Seoul 08826, Korea

^2^Phyzen Genomics Institute, Seongnam, Gyeonggi-do, 13558, Republic of Korea

^3^Department of Horticulture, Chonnam National University, Gwangju 61186, Republic of Korea

^4^Department of Bioresources Engineering, College of Life Sciences, Sejong University, Seoul, 05006, Republic of Korea

+ Both equally contributed.

^*^ Correspondence:

Tae-Jin Yang Tel: +82-2-880-4547; Fax: 82-2-873-2056; e-mail: tjyang@snu.ac.kr

Kihwan Song Tel: +82-2-3408-2905, E-mail: khsong@sejong.ac.kr

**Supplementary Tables**

**Supplementary Table S1.** Characterization of cp genomes of parental inbred lines and their reciprocal F_1_ hybrids (Excel file).

**Supplementary Table S2.** Similarity of nucleotide sequences of cp genomes. Whole cp genome sequences were aligned using MAFFT ver. 7 (https://mafft.cbrc.jp/alignment/server/index.html) and then similarity scores among sequences were calculated using BioEdit sw (Excel file).

**Supplementary Table S3**. List of 292 polymorphic sites found in mt sequences of two parental cucumber inbred lines and F_1_ hybrids (Excel file).

**Supplementary Figures**

**
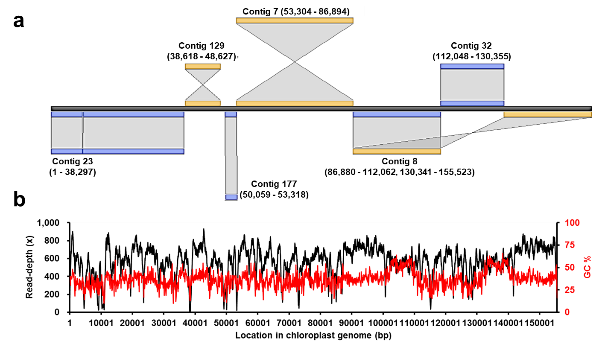
**

**Supplementary Fig. S1.** Assembly example of chloroplast (cp) genome. (A) Alignment of the initial contigs representing the cp genome on the complete cp genome sequence of inbred line MGL. The contig numbers and hit positions are indicated. (B) Status of read mapping on the assembled cp genome sequence. The high-quality PE reads were mapped to the completely assembled cp genome sequence of inbred line MGL (black line). Red line indicates GC content per 100-bp unit length.


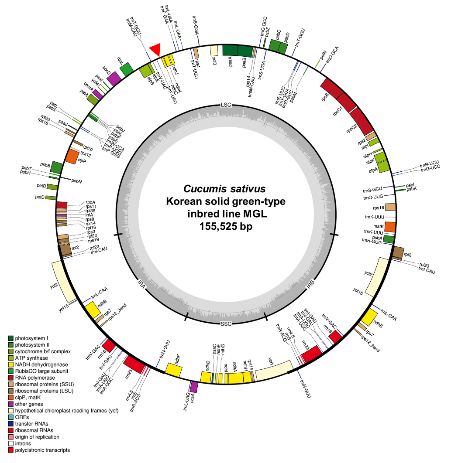


**Supplementary Fig. S2.** Chloroplast genome map of cucumber inbred line MGL. Genes transcribed clockwise and counterclockwise are indicated on the outside and inside of the large circle, respectively. The four parts of the chloroplast genome and GC content are indicated on the inner circle.


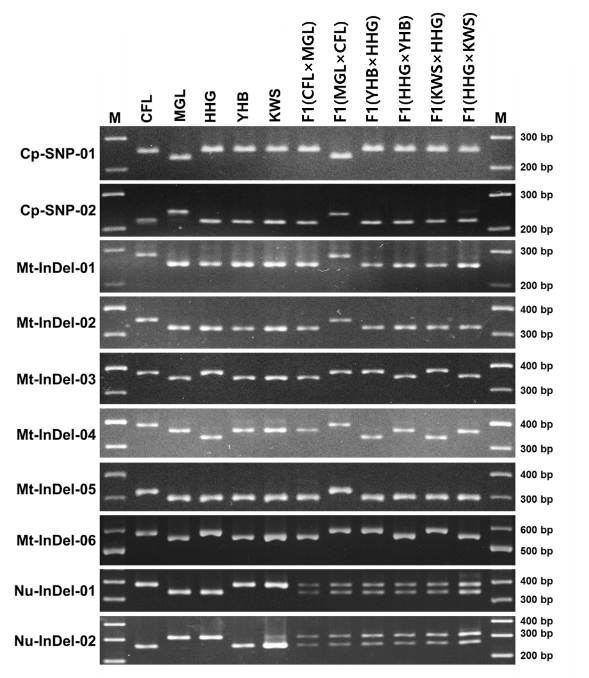


**Supplementary Fig. S3.** Additional validation of molecular markers to confirm inheritance pattern of organelles in cucumber. Eight molecular markers (Cp-SNP-01 to Mt-InDel-06) were designed based on cp and mt sequence polymorphisms and validated using genomic DNA PCR analyses with 11 cucumber samples. Two nuclear InDel markers, Nu-InDel-01 and Nu-InDel-02, were used to confirm heterozygous genotypes of F_1_ hybrid nuclear genomes. M, 100-bp size marker; CFL, a Chinese long green-type inbred line; MGL, a Korean solid green-type inbred line; HHG, a Korean semi-white-type inbred line; YHB, a Korean solid green-type inbred line different from MGL; KWS, a Korean solid green-type inbred line different from MGL; F1(CFLxMGL), an F_1_ hybrid between CFL (maternal) and MGL (paternal); F1(MGLxCFL), an F_1_ hybrid between MGL (maternal) and CFL (paternal); F1(YHBxHHG), an F_1_ hybrid between YHB (maternal) and HHG (paternal); F1(HHGxYHB), an F_1_ hybrid between HHG (maternal) and YHB (paternal); F1(KWSxHHG), an F_1_ hybrid between KWS (maternal) and HHG (paternal); F1(HHGxKWS), an F_1_ hybrid between HHG (maternal) and KWS (paternal). Plant samples used in this analysis were different from those used in Fig. 2.


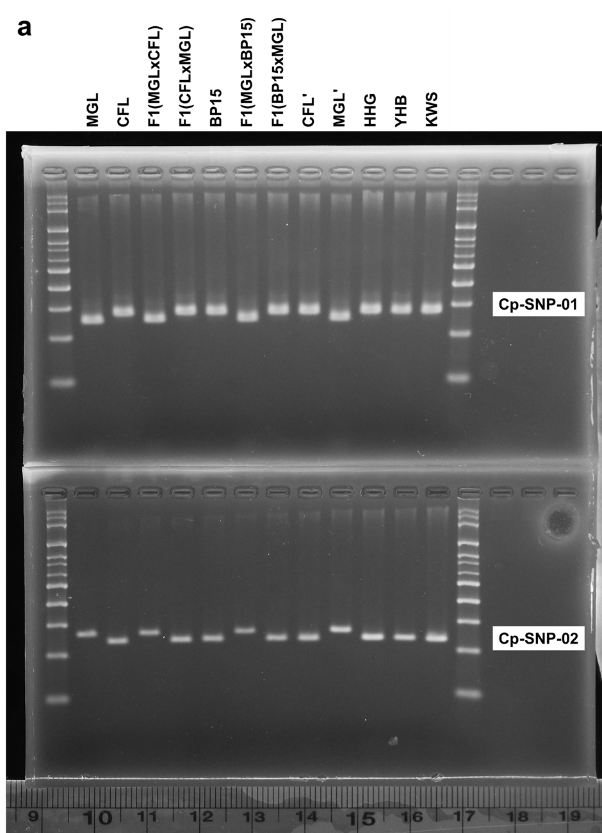


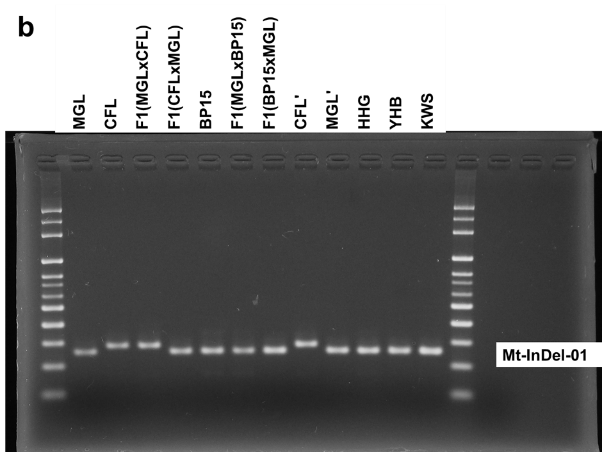


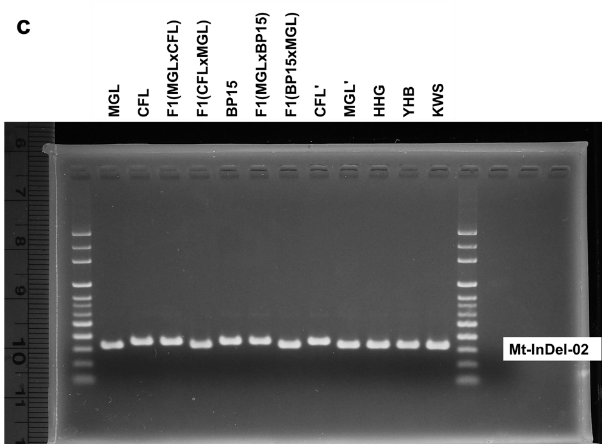


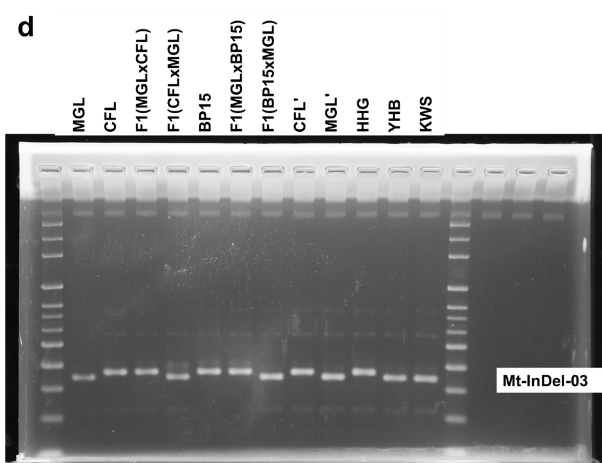


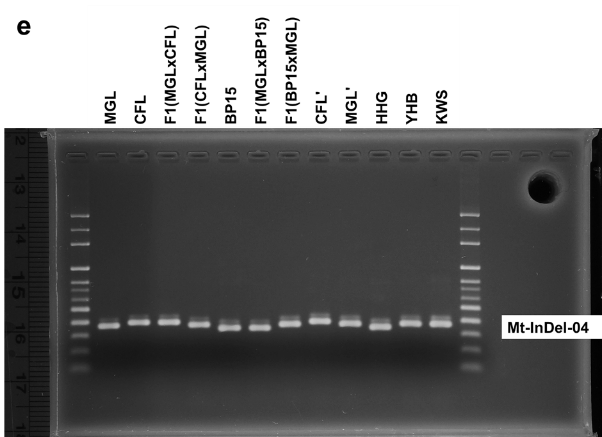


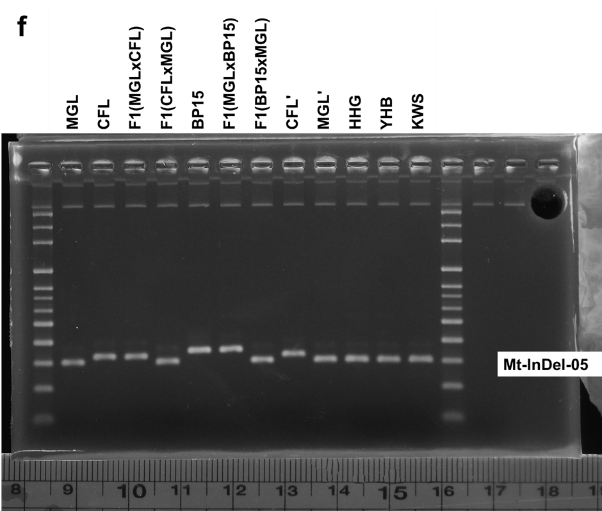


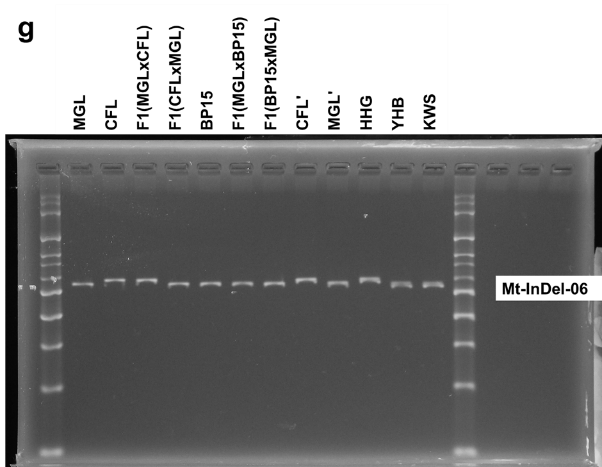


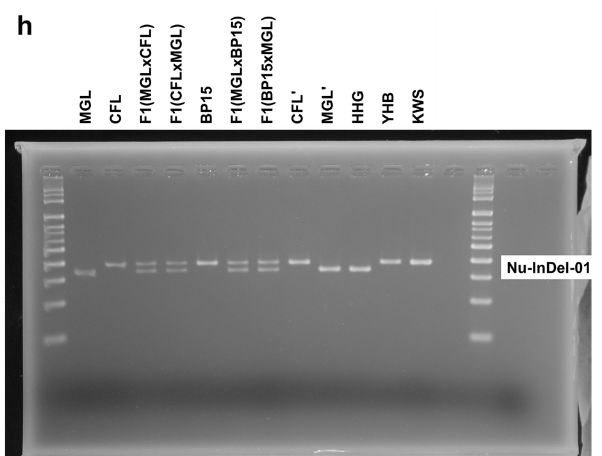


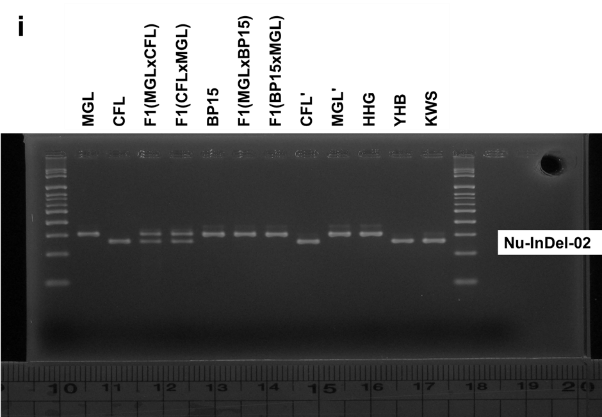


**Supplementary Fig. S4.** Uncropped gel image for Figure 2. a.Cp-SNP-01, b.Cp-SNP-02, c-g. from Mt-InDel-01 to Mt-InDel-06, h. Nu-InDel-01, i. Nu-InDel-02.


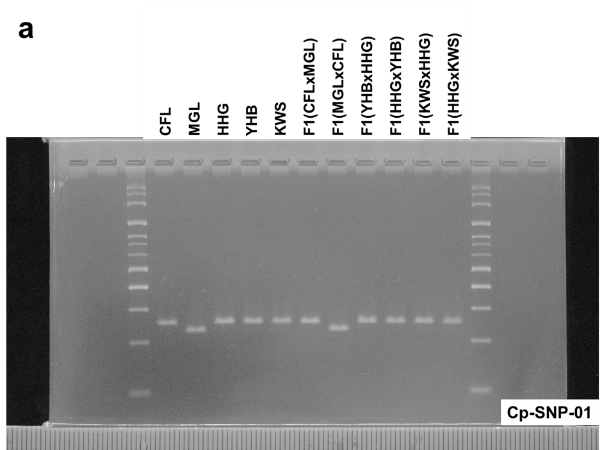


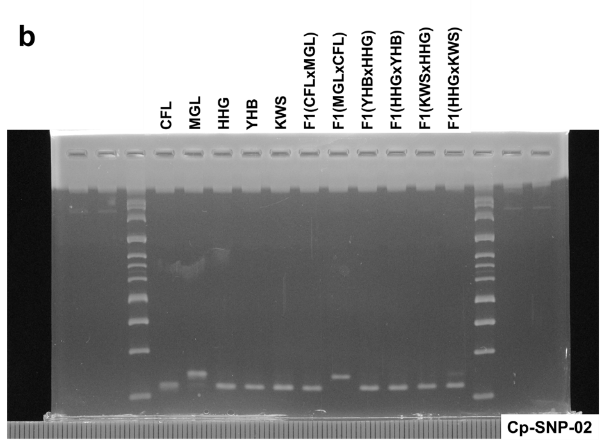


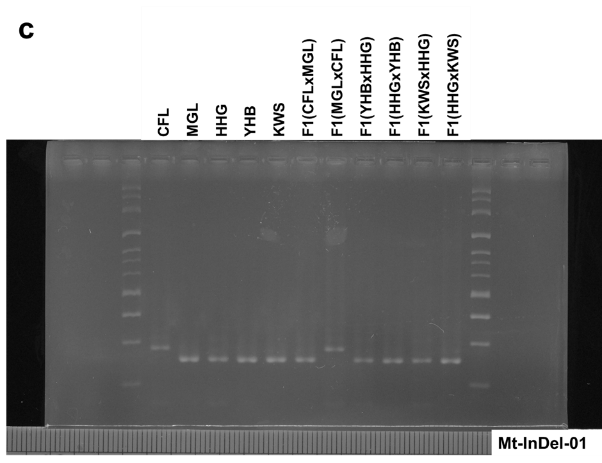


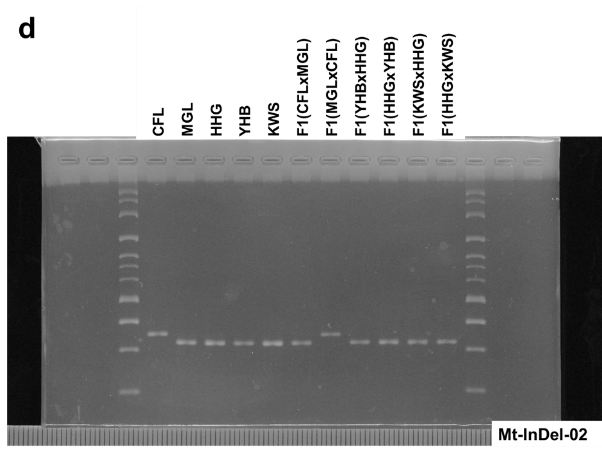


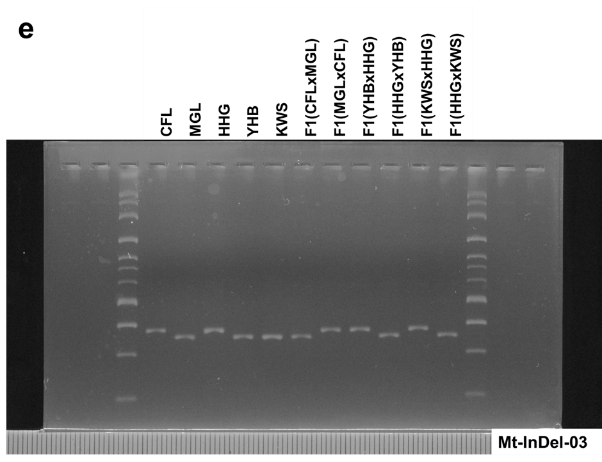


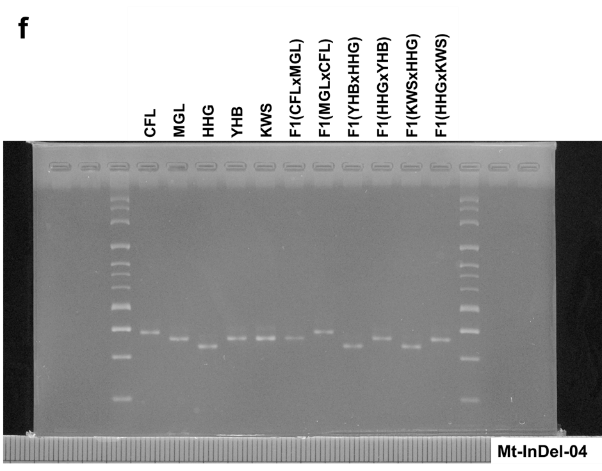


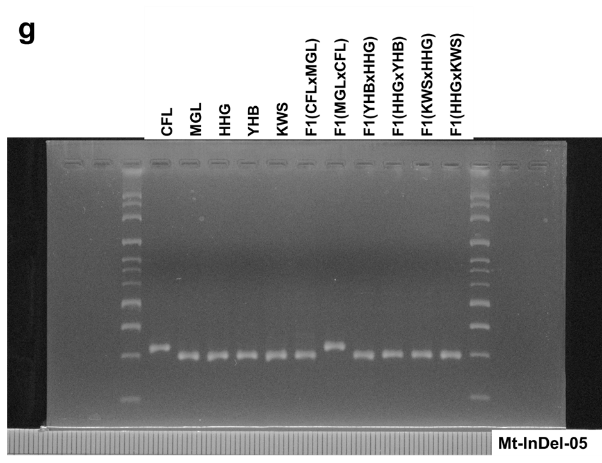


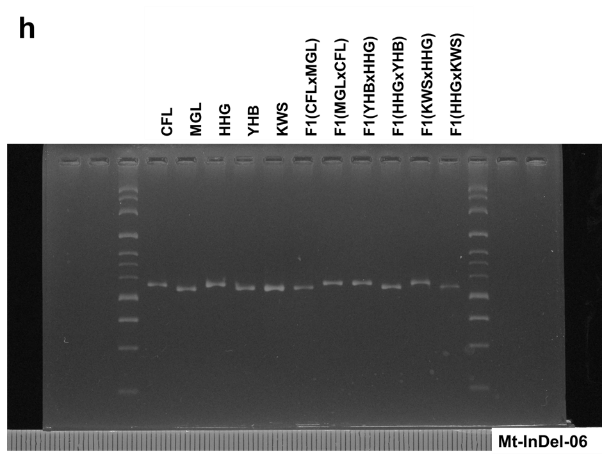


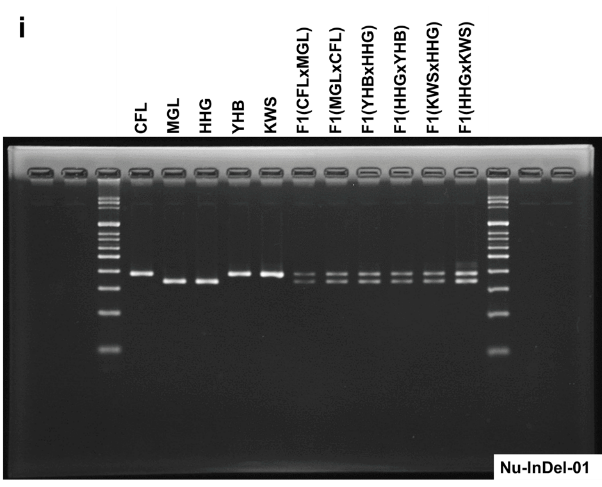


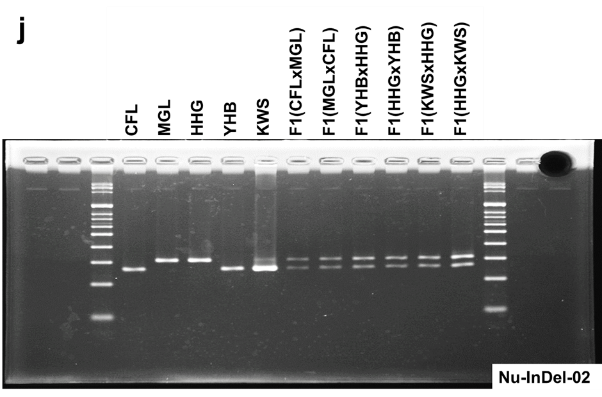


**Supplementary Fig. S5.** Uncropped gel image for Supplementary Fig. S3. a.Cp-SNP-01, b.Cp-SNP-02, c-g. from Mt-InDel-01 to Mt-InDel-06, h. Nu-InDel-01, i. Nu-InDel-02.
